# Freeze-Drying of mRNA-LNPs Vaccines: A Review

**DOI:** 10.3390/vaccines13080853

**Published:** 2025-08-12

**Authors:** MD Faizul Hussain Khan, Floriane Baudin, Ayyappasamy Sudalaiyadum Perumal, Amine A. Kamen

**Affiliations:** 1 Viral Vectors and Vaccines Bioprocessing Group, Department of Bioengineering, McGill University, Montreal, QC H2X 1Y4, Canada; md.f.khan@mail.mcgill.ca (M.F.H.K.); floriane.baudin@mail.mcgill.ca (F.B.); 2 Department of Bioengineering, McGill University, Montreal, QC H3A 0E9, Canada

**Keywords:** mRNA vaccines, mRNA-LNPs, LNPs, formulations, lipid nanoparticles, freeze-drying, lyophilization, vaccine stability, bioprocessing, instability

## Abstract

The instability of mRNA vaccines presents significant challenges for their storage, transportation, and large-scale distribution, particularly in resource-limited countries. Recently, freeze-drying (lyophilization) has been considered as a promising approach for preserving mRNA vaccine efficacy. This formulation technique enhances the long-term stability of mRNA vaccines by converting them into a stable dry powder. The purpose of this review is to provide an overview of the current knowledge on the progress of freeze-drying techniques for mRNA vaccines, with emphasis on the associated challenges. This review highlights the factors influencing the stability of freeze-dried mRNA vaccines and provides a comprehensive overview of the formulation components, including excipients, buffers, and surfactants, as well as the process parameters and storage conditions that aim to improve stability and shelf-life. By providing these insights, this review supports the advancement of more robust, scalable, and efficient lyophilization protocols, ultimately addressing the stability limitations of mRNA vaccines and enhancing their global accessibility.

## 1. Introduction

Messenger RNA (mRNA) vaccines consisting of mRNA encapsulated in lipid nanoparticles (LNPs) have emerged as a rapidly scalable platform in modern vaccinology. mRNA vaccines are now the first choice for treating and preventing a wide range of life-threatening diseases. The scope covers infectious diseases [1,2], cancers [3,4], immunological diseases [5], tissue damage [6], and rare diseases (cystic fibrosis, amyloidosis, and type I diabetes) [7], demonstrating the versatility and potential of mRNA vaccines in diverse therapeutic areas. To date, numerous phases of clinical trials using mRNA vaccines are being successfully conducted against severe acute respiratory syndrome coronavirus 2 (SARS-CoV-2), zika virus, human immunodeficiency virus (HIV), influenza virus, cytomegalovirus, respiratory syncytial virus, varicella-zoster virus, and rabies virus [8,9]. More recently, clinical trials study the efficacy and technical feasibility of mRNA immunotherapeutic against different types of cancers like melanoma [3], brain [10], lung (NSCLC) [11], ovarian [12], prostate [13], hematological [14], digestive system [15], and breast [16] cancer types.

While mRNA vaccines have emerged as a highly promising and effective technology for the prevention of COVID-19, their widespread adoption is limited by their poor thermostability [17,18]. Indeed, one of the primary challenges in the global distribution of mRNA vaccines is their limited thermostability, which requires ultra-cold storage conditions to maintain stability. For instance, the Pfizer-BioNTech COVID-19 vaccine requires storage at −80 °C, whereas the Moderna COVID-19 vaccine requires storage at −20 °C, both with a shelf life of up to 6 months [18,19]. Furthermore, the two recently released COVID-19 mRNA vaccines, SpikeVax and Comirnaty, have reported stability at ambient temperature for only 12 h and 6 h, respectively [20]. Despite several years of COVID-19 mRNA vaccination, the stability issues persist, with limited improvements in the mRNA vaccine product stabilities and storage conditions. Such storage limitations pose significant logistical challenges, particularly in low-and middle-income countries with limited infrastructure, which hinders the widespread use of mRNA vaccines. Therefore, it is crucial to improve the stability of mRNA vaccines at higher temperatures to facilitate their storage, transportation, and distribution.

Building on the foundation and theory of other vaccine formulations, such as viral-vector vaccines, freeze-drying has also been considered a superior method for mRNA vaccines to extend their shelf life [21,22]. LNP formulations use self-assembled polymeric materials that serve as delivery vehicles for nucleic acid payloads, similar to the enveloped viral vector vaccines. These similarities help to extrapolate the freeze-drying process conditions and formulations from viral vector vaccines to mRNA vaccines. Freeze-drying has been used for the stabilization of biological molecules, including enzymes, antibodies, and antigens, for many months and years. For mRNA vaccines, the challenge lies in preserving the LNPs-encapsulated mRNA, which shields the fragile mRNA from physical and chemical agents of degradation. The freeze-drying process, which removes water by sublimation under vacuum at low temperatures, stabilizes the mRNA-LNPs by immobilizing them in a solid matrix of a cryoprotectant. Water removal by sublimation contributes to removing moisture and preventing hydrolysis and oxidation, two common mechanisms of mRNA degradation in liquid formulations [23]. In fact, in a dehydrated state, hydrolysis is less likely to occur, and molecular mobility is drastically reduced, thereby slowing down degradation reactions. Significant research has been conducted in recent years to optimize the freeze-drying process of preventive mRNA vaccines, and several studies have shown promising results.

Furthermore, currently, there is a lack of US FDA regulatory standards specific to lyophilized mRNA-lipid nanoparticle (LNPs) formulations. While general guidelines for biologics, injectable drugs, and liposomal formulations provide a regulatory framework, they do not fully address the unique challenges posed by lyophilizing mRNA-LNPs, such as preserving mRNA integrity, maintaining nanoparticle stability, and ensuring efficient reconstitution. This regulatory gap creates uncertainty in the development and approval process. This review aims to address that gap by pointing out the critical aspects of freeze-drying mRNA-LNPs freeze-dried formulations. Based on the current scientific findings, the review provides an information framework on key parameters such as particle size, polydispersity, encapsulation efficiency, mRNA integrity, and reconstitution behavior that are essential for regulatory assessment but not yet fully adopted.

Overall, the literature review provides a comprehensive overview of the state-of-the-art freeze-drying strategies for mRNA vaccines, focusing on process optimization, methodologies, and advancements in the stabilization of mRNA vaccines. This review also provides an overview of the key principles and challenges associated with freeze-drying mRNA vaccines. Finally, we highlight potential future directions to address the remaining hurdles and facilitate the widespread adoption of freeze-dried mRNA vaccines.

## 2. Stability of mRNA Vaccines

The stability of an mRNA vaccine is influenced by three main factors: (i) the molecular structures of lipid components and their interactions with the mRNA payload, (ii) the excipient formulations (e.g., buffers and cryoprotectants) and (iii) formulation techniques (e.g., freeze-drying cycles) as well as the storage conditions.

### 2.1. Structure and Delivery Mechanism of mRNA Vaccines

**Figure 1:**
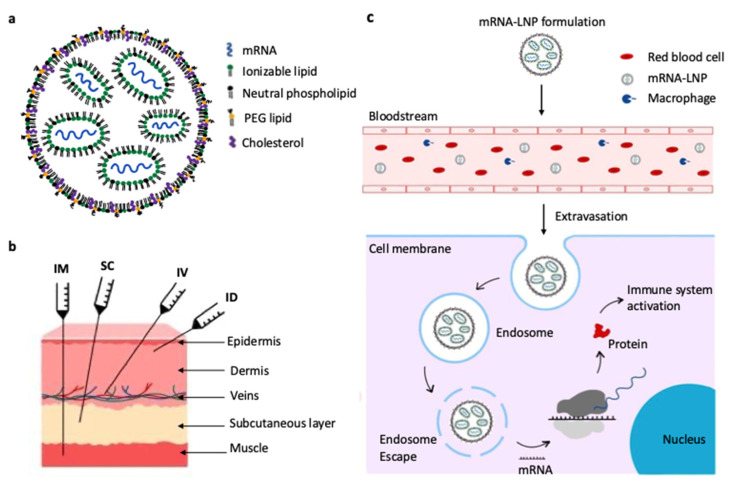
Structure, methods of vaccine administration and mechanism of cellular uptake of mRNA vaccines. (**a**) Lipid nanoparticle (LNPs) composition illustrating ionizable lipid, PEG-lipid, cholesterol, and phospholipid surrounding encapsulated mRNA. (**b**) Administration routes of mRNA-LNPs, including intramuscular (IM), subcutaneous (SB), intravenous (IV), and intradermal (ID) injection. (**c**) Ideal expectation of cellular uptake via endocytosis, endosomal escape facilitated by ionizable lipids, and mRNA release into the cytoplasm for translation.

LNPs for mRNA-based vaccines offer several advantages, including ease of formulation [24], modularity [2], biocompatibility [25] and a flexible capacity for mRNA payload (1000 to 10,000 nt) [25]. LNPs are typically composed of four lipids: an ionizable lipid, a polyethylene glycol-functionalized lipid (PEG-lipid), an amphiphilic phospholipid, and cholesterol (Figure 1a). The ionizable lipid encapsulates negatively charged mRNA at low pH during LNPs assembly, thereby promoting further endosomal release via protonation in the acidic endosomal environment (pH 4.5–6.8) [26,27]. The other lipid components improve the LNPs properties, including stability, delivery efficiency, tolerability and biodistribution. Cholesterol enhances the LNPs stability by filling in the gaps between the phospholipids, thereby influencing the membrane integrity and rigidity [28]. The PEG-lipid regulates particle size, and zeta potential further enhances the particle stability by forming a steric barrier against aggregation [29]. Further, phospholipids increase mRNA encapsulation efficiency and promote fusion with cellular and endosomal membranes, thereby facilitating cellular uptake and endosomal release [30]. Specifically, phospholipids with high transition temperatures, such as 1,2-distearoyl-*sn*-glycero-3-phosphocholine (DSPC, transition temperature of 55 °C), can stabilize the LNPs structure by forming a lamellar phase, thus increasing membrane rigidity and reducing permeability [31].

Figure 1b shows the various delivery routes for mRNA-LNPs through injection. These include intramuscular (IM), subcutaneous (SC), intravenous (IV), and intradermal (ID) routes. Each method targets different layers of tissue. These different routes allow for specific immune responses based on the vaccine goal. These administration routes influence the vaccine’s behavior in the body and the subsequent immune response [22,32].

After administration of mRNA-LNPs, the nanoparticles enter the bloodstream and circulate alongside red blood cells and immune cells (Figure 1c). Through extravasation, the LNPs exit the bloodstream and penetrate the target tissues. Upon encountering a cell, the LNPs are internalized via endocytosis and form endosomes [33]. Inside the cell, the ionizable lipids become positively charged in the acidic environment of the endosome. This disrupts the endosomal membrane and releases the mRNA into the cytosol [34]. The mRNA escaping the degradation pathways is translated by the cell’s ribosomes to produce the encoded protein. In the context of vaccination, this protein acts as an antigen that stimulates an immune response. Thus, it triggers the immune system to recognize and combat the respective diseases [8,32,33,34,35].

Despite the protective encapsulation, the mRNA payload remains inherently unstable due to the presence of its reactive 2′-hydroxyl (2′-OH) groups on the nucleic acid bases, causing spontaneous cleavage (especially under heat, alkaline pH, or in the presence of metal ions) [36]. LNPs are also sensitive to physical stress, especially during freeze-drying and subsequent rehydration [37]. Several studies concluded that mRNA hydrolysis is a key determinant of mRNA-LNPs instability. These studies improve mRNA stability by optimizing mRNA nucleotide composition and structure [36,38].

**Figure 2:**
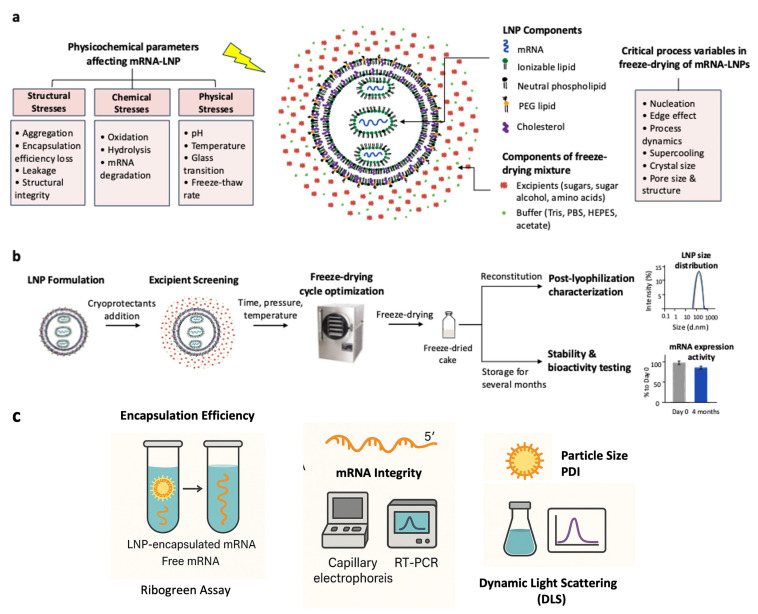
Key Considerations and Workflow for Developing Freeze-dried mRNA Vaccine Formulations. (**a**) Schematic representation of mRNA-loaded lipid nanoparticles (LNPs) highlighting physicochemical stresses affecting stability (structural, chemical, and physical) and key critical process variables to control during freeze-drying. (**b**) Stepwise process for the development and evaluation of freeze-dried mRNA vaccines. (**c**) To evaluate the mRNA-LNPs stability over time, the RiboGreen assay is used to measure the encapsulation efficiency, capillary electrophoresis or RT-PCR are used to detect the mRNA integrity at the 5′ and 3′ ends, and DLS is used to assess the LNPs structure via particle size and zeta potential.

In addition to the active pharmaceutical ingredient (LNPs-encapsulated mRNA), LNPs consist of several self-assembled components that can interact dynamically with each other, thereby influencing the overall stability of the formulated product (Figure 2). For instance, the major degradation mechanisms for lipids in the LNPs is oxidation that occurs due to exposure to light, oxygen, metal residues, and high temperatures [37]. Hydrolysis also represents an important degradation mechanism influenced by lipid structure, pH, temperature, and buffer composition (Figure 2). For example, amino lipids, such as those used in the Moderna and Pfizer/BioNTech mRNA-LNPs COVID-19 vaccines, can generate reactive electrophilic impurities. This inactivates the mRNA through N-oxidation and hydrolysis and forms reactive aldehydes that adversely affect biological performance [19,36,39].

Physical instability (Figure 2) can be characterized by the aggregation or fusion of the LNPs, which manifests as an increase in particle size or polydispersity index of the LNPs [18]. Chemical instability is most often observed as degradation of the mRNA payload and/or lipid components [18]. Thus, both forms of instability impose challenges for storage in an aqueous buffer as a wet or liquid formulation. Extrinsic parameters, such as the pH of the storage buffer and temperature, can also affect stability. Molecular mobility in a liquid formulation can impact the LNPs stability during storage under liquid conditions [36,40]. The addition of excipients helps reduce molecular mobility during ambient or refrigerated storage conditions. However, these methods are not effective; therefore, sub-zero storage at −20 °C and −80 °C have been used [36]. As a result, lyophilization appears to be a promising solution to improve the stability of mRNA-LNPs vaccines by converting wet formulations into more stable dry formulations, thus reducing molecular mobility [31,41].

### 2.2. Stabilizing mRNA Vaccines Through Freeze-Drying

Due to the poor long-term stability of liquid mRNA vaccines and their limited shelf life at non-frozen temperatures, freeze-drying has gained attention as a strategy to enhance thermostability, with promising results reported in recent studies. Kim et al. [42] reported that mRNA-LNPs composed of TT3, Dlin-MC3-DMA, DOPE, cholesterol, and DMG-PEG2000 at a molar ratio of 10:25:20:40:5, and stabilized with 10% (*w*/*v*) sucrose, remained stable for at least 30 days at −20 °C. Additionally, Muramatsu et al. [43] synthesized freeze-dried mRNA-LNPs composed of PEG lipid (PEG2000-C-DMA), cholesterol, DSPC, and an ionizable lipid ((6Z,16Z)-12-((Z)-dec-4-en-1-yl) docosa-6,16-dien-11-yl 5-(dimethyl amino pentanoate) in a molar ratio of 1.5:38.5:10:50, encapsulating firefly luciferase (Fluc)-encoding mRNA (101-nt-long, poly-A tails). They also demonstrated that stability during storage was dependent on the storage temperature over a wide range of temperatures, specifically −80, −20, 4, 25, and 42 °C. In another study, lyophilized formulations containing 10% (*w*/*v*) sucrose and 10% (*w*/*v*) maltose as cryoprotectants preserved stability and physicochemical integrity for over 12 weeks at room temperature and at least 24 weeks at 4 °C [44]. Similarly, Wang et al. [31] found that using an optimal 8.7% (*w*/*v*) concentration of C-type mRNA-LNPs ensured stability for more than 12 weeks at 4 °C. In a recent study, Alejo et al. [45] demonstrated that by optimizing the buffer system (favouring Tris over PBS), selecting effective cryoprotectants (such as sucrose or maltose), and fine-tuning the freeze-drying parameters, the lyophilized LNPs retained in vivo bioactivity level for 1 year when stored at 4 °C. In vivo bioactivity refers to the biological effect of a vaccine, protein, or therapeutic agent when tested in an animal model, such as a mouse or rat. The research group also demonstrated that lyophilized LNPs retained their thermostability at room temperature for 4 weeks. To evaluate the stability of their formulations, the authors employed dynamic light scattering (DLS), zeta potential analysis, mRNA encapsulation efficiency (Ribogreen) assays, in vitro transfection experiments in HeLa and 293T cells, and in vivo bioluminescence imaging in animal models [45].

Additionally, Shirane et al. [46] described a novel approach for freeze-drying mRNA-LNPs that retains ethanol in the formulation, known as the “alcohol–dilution–lyophilization method.” Originally, the method was successfully applied to siRNA-loaded LNPs. This one-pot process combines two key steps of alcohol dilution and freeze-drying into a single workflow. By avoiding ethanol removal, this method simplifies production and reduces the number of processing steps. Instead of removing ethanol, the mRNA-LNPs were directly freeze-dried, resulting in a solid formulation stable at 4 °C for at least 4 months.

Overall, these freeze-dried formulations demonstrated a significant improvement in stability over time compared to liquid formulations. Freeze-drying of mRNA vaccines offers the advantages of enabling ambient storage compared to sub-zero temperatures, as well as extending shelf life, which facilitates storage, transportation, and distribution. However, the freeze-drying process is complex and requires fresh screening of excipients, optimization of reagent and process parameters, such as buffer, excipient, and surfactant concentrations, cycle duration, and iterations, as well as temperature, to preserve LNPs physicochemical properties (Figure 2). Further research into the development of stabilizers and optimization of freeze-drying protocols is essential to enhance the thermostability of all mRNA vaccine formulations.

### 2.3. Challenges During Freeze-Drying of mRNA Vaccines

**Figure 3:**
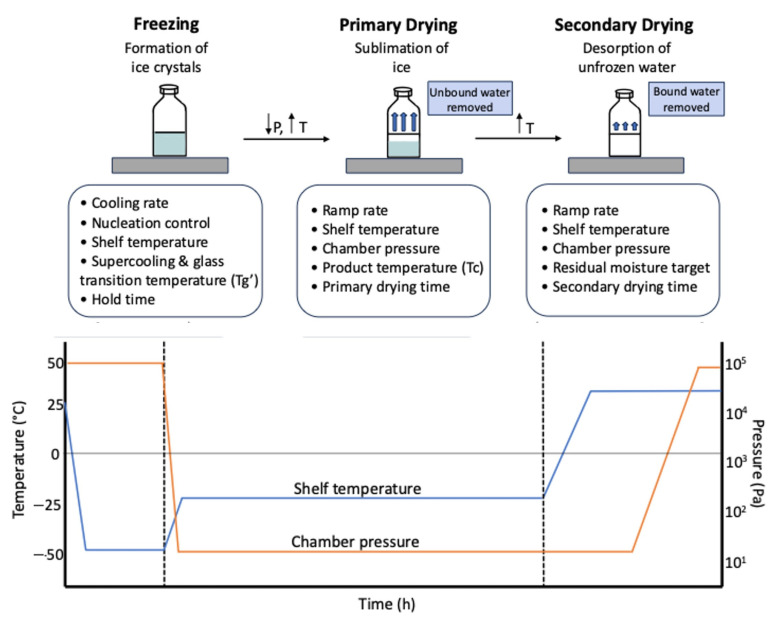
Critical Process Parameters Across the Freeze-drying Cycle of mRNA Vaccines.

The freeze-drying process involves three stages: freezing, primary drying (sublimation), and secondary drying (desorption) (Figure 3). Each stage presents unique challenges for mRNA vaccines, affecting the preservation of the LNPs structure and the stability of the encapsulated mRNA. For instance, freezing can lead to the formation of ice crystals that disrupt the LNPs membrane, while drying stages can introduce mechanical stress and dehydration-induced destabilization of lipid components. Both the freezing phase and drying phase are known to induce stresses to lipid-based nanoparticle formulations [47]. Alejo et al. [45] demonstrated that freezing and dehydration impose mechanical stress and deformation on lipid structures, leading to LNPs aggregation and the release of encapsulated mRNA (Figure 2). During freezing, the formation of ice crystals can damage the LNPs, potentially leading to aggregation or leakage of mRNA. Careful optimization of this phase is crucial to ensure product quality and transfection efficiency. During the secondary drying phase, improper control of drying temperatures and pressures can also lead to irreversible damage to the lipid bilayer.

## 3. Formulations

During lyophilization, mRNA-LNPs is exposed to a variety of stresses like pH changes, freeze concentration, and aggregation caused by drying (Figure 2) [36,43]. Therefore, an adequate formulation must be used to protect the product and ensure its integrity and activity. The development of lyophilization protocols must consider both formulation and process optimization simultaneously to establish an effective process for freeze-drying mRNA vaccines [48]. In this section, different stabilizers commonly used in mRNA-LNPs formulations are described, and their influence on the freeze-drying process is discussed.

### 3.1. Influence of Lipid Composition

The choice of lipids and composition and specific components of the lipid nanoparticles have specific functions during particle formation, such as stabilization and biological performance, and it is critical to maintain the pharmacologically active drug product. Wang et al. [31] showed that the DSPC/cholesterol ratio might be the most influential factor for the stability and transfection efficiency of mRNA-LNPs after lyophilization. The authors found that ratios of DSPC to cholesterol ranging from 2 to 2.2 appeared to be most suitable for maintaining the high transfection efficiency of mRNA-LNPs after lyophilization. Using HEK-293T cells, they found that the ionizable lipids critically determined the transfection efficiency, and a further important ingredient was the PEGylated lipid used in the mRNA-LNPs.

### 3.2. Stabilizers

To address challenges associated with the inherent sensitivity of mRNA to environmental stressors during the freeze-drying process, various stabilizers have been incorporated into the formulation of freeze-dried mRNA vaccines. Stabilizers are added to protect the mRNA-LNPs against chemical and physical degradation during processing and storage. Selecting an appropriate stabilizer is key to protecting the mRNA-LNPs against structural changes, fusion/aggregation, membrane damage caused by ice crystals, intracellular ice formation and pH shift [21,49]. Different stabilizers, also known as cryoprotectants, have been used to stabilize freeze-dried mRNA vaccines.

Common stabilizers include sugars like sucrose and trehalose, sugar alcohols such as mannitol and sorbitol, and amino acids like histidine. As the exact stabilization mechanisms for mRNA vaccines remain unclear, published studies have identified valuable trends that guide the formulation strategies [50,51].

#### 3.2.1. Sugars

Sugars such as trehalose and sucrose are commonly used in formulations due to their ability to protect various types of vaccines and biopharmaceutical products (Table 1 and Table 2) [52]. They are described as very effective stabilizers in multiple studies, as they exhibit high viscosity, low molecular mobility after drying, and form an amorphous glassy matrix [21]. Recent studies by Kefetzis et al. [51] and Zhang et al. [53] have demonstrated that incorporating these stabilizers into formulations can significantly enhance the long-term stability of mRNA-LNPs, allowing for effective storage at ambient temperatures. Although not definitively confirmed, sucrose and trehalose are thought to protect mRNA-LNPs during freeze-drying through vitrification and/or hydrogen bonding (water substitution), helping to prevent stress-induced damage during the process [21].

The presence of sucrose not only prevents the aggregation of LNPs but also protects the mRNA from freezing and dehydration stresses that could lead to a loss of potency [21]. Sucrose is used in concentrations ranging from 2 to 20% (*w*/*v*), as higher concentrations may result in difficulties in reconstitution of the formulation after lyophilization [31,42]. For instance, Wang et al. [31] demonstrated that 8.7% sucrose is the optimal cryoprotectant concentration to maintain the transfection efficiency of lyophilized mRNA-LNPs, and Kim et al. [29] found that mRNA vaccines were stably stored at −20 °C for at least 30 days when 10% (*w*/*v*) sucrose was added to PBS. Kim et al. [42] also showed that for both PBS (phosphate-buffered saline)- and TBS (Tris-buffered saline)-stored LNPs, the absence of sugar from the buffer resulted in a 20–50 nm increase in the particle size distribution, hinting at the cryoprotective effects of sucrose.

Sucrose is one of the most used cryoprotectants in mRNA vaccine formulations, as it effectively stabilizes LNPs during the freeze-drying process (Table 2). However, there is no single optimal concentration or percentage for LNPs formulations. Additionally, the percentages of sugars as excipients in the LNPs formulation may vary based on several parameters, such as the composition of the LNPs particles, payload sizes, and whether the cargo is single or dual (Table 2).

#### 3.2.2. Sugar Alcohols

Mannitol is a frequently used sugar alcohol in mRNA-LNPs formulations, serving primarily as an excipient in spray-freeze drying or as a bulking agent during lyophilization (Table 1) [61]. Its addition in freeze-dried products helps form a crystalline phase instead of an amorphous one, which helps prevent cake shrinkage. For example, Luo et al. [50] demonstrated that replacing disaccharides, which remain amorphous after freeze-drying, with mannitol provided the necessary crystallinity to maintain the structural integrity of the lyophilized cake, thus preventing cracking. Mannitol has also been shown to improve the stability of LNPs during freeze-drying [42].

Since mannitol is the most commonly used sugar alcohol in the excipient formulation, there exists a possibility to explore new types of sugar alcohols for formulation applications.

#### 3.2.3. Amino Acids

Excipients such as arginine, histidine, glycine, and methionine are deemed to be commonly used amino acid stabilizers. These amino acids stabilize the formulations through preferential hydration, exclusion, and enhanced solubility. Although arginine, glycine and methionine are commonly used as stabilizers in protein formulations, histidine is also used in mRNA-LNP formulations, particularly as a buffer at pH values between 5.0 and 7.0. Histidine has been linked to low-viscosity formulations, making it an attractive excipient [69].

Currently, histidine is the closest usable amino acid in the LNPs formulation context; however, there is scope for other amino acid stabilizers, which require further studies

### 3.3. Influence of pH and Buffer

Besides the choice of stabilizers, the choice of buffer and pH can also significantly impact the thermostability of mRNA vaccines (Table 1). In 2021, the Comirnaty^®^ vaccine formulation was updated by replacing the originally used PBS (phosphate-buffered saline) with Tris (short for tris (hydroxymethyl) aminomethane) as the buffering agent, which enhanced its stability, gaining approval from the Committee for Human Medicines (CHMP) at the European Medicines Agency (EMA) for storing the BioN- Tech/Pfizer vaccine between −15 and −25 °C for up to two weeks [45]. Recent works, aligned with the buffer used for Comirnaty, suggested that buffer composition can influence the ionic strength, pH stability, and interaction with lipid and nucleic acid components, ultimately affecting the physical and chemical stability of mRNA-LNPs formulations [60].

Indeed, buffer composition not only affects the ionic environment but also helps mitigate pH fluctuations during freezing, which can significantly influence the rate of mRNA hydrolysis [70]. Phosphate buffers are known to undergo substantial pH changes upon freezing, with drops of up to 3.6 units when cooled from 0 °C to −30 °C, whereas Tris and histidine buffers exhibit only minor pH shifts (~0.5–0.6 units) [64,71]. These pH shifts can accelerate mRNA degradation, particularly under acidic conditions and in the presence of divalent cations such as Mg^2+^ and Ca^2+^ [72]. In contrast, Tris-HCl helps stabilize nucleic acids by maintaining pH and scavenging hydroxyl radicals, thereby improving the chemical stability of mRNA during storage and lyophilization [63]. For instance, Alejo et al. [45] demonstrated that 5 mM Tris (pH 7.4) is more efficient than PBS (pH 7.4) at preserving the physicochemical and functional properties of mRNA-LNPs during freeze-drying, regardless of the cryoprotectants used. The particle size and zeta potential were maintained within limits after lyophilization, indicating the choice of buffers contributed to the stability of the formulations.

In contrast, the use of PBS buffer for mRNA-LNPs stabilization resulted in reduced encapsulation efficiency, a marked increase in particle size, and a shift toward a more negative zeta potential [45]. In another study, Henderson et al. [62] demonstrated that Tris and HEPES buffer yielded better cryoprotection and transfection efficiency for LNPs stored frozen at −20 °C compared to PBS. The superiority of Tris buffer compared to PBS was also demonstrated by Meulewaeter et al. [47], who evaluated the impact of three different buffers, including Tris, PBS, and phosphate buffer, on the freeze-drying of mRNA-LNPs. It was found that the properties of mRNA-LNPs dispersed in phosphate and Tris buffer remained unchanged upon lyophilization and that the mRNA-LNPs retained their transfection efficiency. In contrast, a loss in encapsulation efficiency and a drastic decrease in transfection efficiency were observed when using PBS. Therefore, they concluded that phosphate and Tris, but not PBS, were appropriate buffers for the lyophilization of mRNA-LNPs, aligning with the findings of other studies. Finally, Fan et al. [73] conducted the high-throughput screening of 45 cryoprotectants and buffer conditions for mRNA-LNPs. They identified PVP-K12 in Tris or acetate as the optimal cryoprotectant/buffer combinations to maintain the physical stability of mRNA-LNPs, resulting in both minimal particle size increase and minimal decrease in encapsulation efficiency post-lyophilization. It was found that 5–20% PVP-K12 in Tris or acetate buffers achieved both the physical stability of mRNA-LNPs and the chemical stability of the encapsulated mRNA, suggesting that Tris and acetate are better buffers than PBS [73].

Additionally, HEPES has been shown to offer advantages over PBS in certain cases. Specifically, Henderson et al. [62] reported that HEPES-buffered saline (HBS) outperformed PBS and Tris-buffered saline (TBS) in preserving the properties of LNPs after freeze-thaw cycles. While both TBS and HBS buffers enhanced transfection efficiency compared to PBS, HBS provided additional protection against pH shifts and aggregation [62].

Overall, HEPES, along with Tris, appears to be a more suitable buffer than PBS for maintaining the stability and functionality of mRNA-LNPs during storage and lyophilization [67,73].

### 3.4. Impact of Reconstitution Buffer

The reconstitution process can also influence the stability of freeze-dried mRNA-LNPs products. While most mRNA-LNPs formulations are reconstituted in water, some studies have employed buffers for reconstitution (Table 2). Meulewaeter et al. [47] evaluated the impact of different reconstitution buffers by using 400 μL of Tris, phosphate, or PBS buffer at pH 7.4 to reconstitute lyophilized cakes immediately after the freeze-drying cycle. The properties of the rehydrated mRNA-LNPs, including size, polydispersity index (PDI), zeta potential, and mRNA encapsulation efficiency, were analyzed after rehydration. The findings by Meulewaeter et al. [47] indicated that the size of all mRNA-LNPs formulations remained unchanged after lyophilization. However, encapsulation efficiency and the amount of mRNA encapsulated decreased when reconstituted in PBS, while phosphate and Tris buffers preserved the mRNA-LNPs properties mentioned above. The points mentioned above highlight that the choice of buffer for reconstitution can impact the characteristics of the freeze-dried product.

Several studies have used buffer-free formulations, that is, the reconstitution after freeze drying used RNase, DNase, pyrogen-free, sterile, distilled water (Table 2). In fact, buffer-free approaches are often preferred to avoid additional excipients that could cross-react with the freeze-dried LNPs, leading to variations in the final formulations and even other complications. For instance, histidine in the reconstitution buffer can cause discoloration, and citrate can be associated with a stinging sensation when injected subcutaneously or may lead to toxic effects due to the chelation of calcium in the blood [74]. Acetate in the reconstitution buffer can sublime during freeze-drying, which may limit its usefulness in lyophilization. Some buffers, such as phosphate, can undergo selective crystallization during cooling, resulting in significant pH shifts. In addition, Tris has a high Δp*K*_a_/°C; its pH shifts from pH 7.1 at 25 °C to pH 5.0 at 100 °C [74].

## 4. Lyophilization Process Development and Intensification

The freeze-drying process itself must be optimized to ensure the stability of mRNA vaccines. Factors such as the freezing rate, primary drying temperature, and final moisture content are critical parameters that influence the stability and quality of the final product. The development of a lyophilization cycle must be tailored to the specific formulation; therefore, the temperature, pressure, and time conditions must be optimized for each new product. This optimization is essential due to the impact that these parameters have on product critical quality attributes, including mRNA integrity, encapsulation efficiency, particle size (~80–100 nm), PDI, surface charge, lipid composition, transfection efficiency and immunogenicity.

Like enveloped viral vectors, mRNA vaccines share supramolecular assemblies that encapsulate nucleic acids and are sensitive to freeze-drying stress, including H shifts, phase separation, osmotic imbalance, and ice-induced mechanical disruption [75,76]. As Felix Franks emphasized, the design of a successful lyophilization cycle depends on understanding the thermochemical and thermomechanical behavior of amorphous formulations, particularly their glass transition temperature (Tg), ice crystallization tendencies, and solute interactions. These considerations are equally applicable to mRNA-LNPs, which relies on excipients to maintain the LNPs nanostructure and encapsulated cargo.

Also, this is important to use compatible excipients, such as bulking agents and cryoprotectants, to maintain the structure and activity of the product while keeping residual moisture low to ensure good stability and reconstitution [77]. Although originally developed for proteins and viral vector vaccines, these principles apply well to mRNA-LNPs, which have similar structural sensitivities. For example, excipients such as sucrose and trehalose are commonly used to protect both proteins and mRNA-LNPs by forming a stable glassy matrix. The choice of buffer during reconstitution is also critical for preserving product integrity. By building on these established guidelines, researchers can design more effective freeze-drying protocols for mRNA vaccines, thereby reducing their dependence on empirical methods.

Table 3 presents an overview of the freeze-drying process parameters used in various mRNA vaccine studies. However, comparing the different process conditions (temperature, pressure, and time) between the different studies is difficult because critical product parameters, such as Tg’, Tc, and filling volume, are formulation dependent. Table 3 illustrates a high variability in freeze-drying conditions, particularly in terms of freezing, primary drying, and secondary drying stages, reflecting differences in the optimization strategies employed. Freezing temperatures range from −30 °C to −80 °C with durations spanning 20 min to 12 h. Primary drying conditions also show significant variation, with temperatures from −50 °C to −10 °C and pressures between 6 Pa and 24 Pa, lasting anywhere from 4 to 84 h. Secondary drying stages typically occur at higher temperatures (ranging from 2 °C to 30 °C) and moderate pressures (3 to 20 Pa) for durations of 5 to 10 h. These studies indicate a broad range of approaches, with differences often lying in the specific temperature and pressure settings, which are optimized based on formulation composition, volume, and specific critical quality attributes (CQAs).

## 5. Critical Process Parameters (CPPs) and Critical Quality Attributes (CQAs)

Following freeze-drying with optimized process parameters, a freeze-dried cake is obtained. The appearance of this cake is a subjective yet important quality attribute for assessing the success of the lyophilization process. Ideally, the cake should maintain the same size and shape as the original liquid fill and exhibit a uniform colour and texture [82]. The most critical quality indicator is a smooth, uniform white surface, with no evidence of cake collapse, shrinkage or structural failure [65,83]. Multiple studies focusing on the freeze-drying of mRNA vaccines describe the resultant lyophilized product as a white, fluffy cake [43,65].

Critical process parameters (CPPs) and critical quality attributes (CQAs) are crucial for ensuring the efficacy and reproducibility of mRNA-LNPs products from batch-to-batch production. CPPs refer to those process parameters that are indispensable for a robust production process, with minimum variability of the final product. Some of the CPPs for mRNA-LNPs are lipid composition ratio, ethanol concentration, buffer, pH, total flow rate, and N/P ratio (amine-to-phosphate groups in RNA) during the encapsulation process [84,85]. These CPPs have a direct influence on CQAs like particle size, polydispersity index (PDI), encapsulation efficiency, RNA payload distribution across LNPs, zeta potential, and RNA integrity [36,86,87]. Thus, the CQAs refer to the attributes that are used to quantify the quality attributes.

In some cases, lipid ratios (e.g., ionizable cationic lipids, PEG-lipids) were found to determine the nanoparticle stability and cellular uptake [22,87]. On the other hand, flow rate and pH during microfluidic mixing impact particle size, encapsulation efficiency and distribution of RNA payloads [85,88]. The CPPs and CQAs are further discussed below.

### 5.1. Critical Process Parameters (CPPs)

Among the key CPPs, lipid composition ratio is considered critical; the type and molar ratio of ionizable lipids, helper lipids, cholesterol, and PEG-lipids determine nanoparticle structure, encapsulation efficiency, payload distribution, and stability (Table 4) [47,89]. In the case of microfluidic mixing for encapsulation, the total flow rate, mixing speed, and temperature affect particle size, polydispersity, and encapsulation efficiency. Other critical parameters are buffer (e.g., phosphate or Tris, but not PBS) and pH, which are crucial for maintaining LNPs integrity during both formulation and freeze-drying. Improper buffer selection can lead to aggregation or loss of encapsulation. [45]. Another important parameter is the lipid and mRNA weight ratio, where a sufficiently high ionizable lipid to mRNA weight ratio (3:1) is necessary to maintain the encapsulation efficiency [53].

During the freeze-drying cycle, the freezing temperature, primary and secondary drying temperatures, chamber pressure, and ramp rates are critical [31]. Figure 3 illustrates the importance of precise control over temperature and pressure at each stage. Additionally, the choice and amount of cryoprotectants (e.g., sucrose, trehalose) are crucial for protecting LNPs and mRNA during freezing and drying. They can also affect the cake structure, reconstitution time, and preservation of CQAs [65].

### 5.2. Critical Quality Attributes (CQAs)

Critical quality attributes (CQAs) for freeze-dried samples include the maintenance of LNPs physicochemical structure and integrity before and after lyophilization (Table 4). Among the CQAs, physical characteristics, including the particle size and polydispersity index (PDI), are commonly used. The standard size of the mRNA-LNPs is typically 80–200 nm, with a low polydispersity index (PDI) or high uniformity, which is critical for biodistribution, cellular uptake, and immunogenicity. Both the CQAs should remain stable throughout the freeze-drying process and during subsequent reconstitution. Another important attribute is encapsulation efficiency; a high encapsulation efficiency (≥80%) ensures that the maximum amount of mRNA is encapsulated within the LNPs. High encapsulation efficiency must be maintained throughout freeze-drying and during storage to maintain its efficacy [41,47]. The zeta potential of LNPs typically ranges from slightly positive to near-neutral (around +5 to +20 mV), depending on the formulation conditions and lipid composition. A balanced zeta potential helps optimize cellular uptake by promoting interaction with cell membranes [40]. To be considered an effective vaccine, the integrity and purity of the mRNA need to be well maintained, which is determined by capillary electrophoresis or similar methods. Lyophilization or storage conditions should maintain the integrity of the mRNA. Additionally, the lipid composition is crucial for ensuring the efficient uptake of mRNA by the cells.

Most importantly, potency and transfection efficiency are the key CQAs for antigen expression, whether in vitro or in vivo. These attributes must be retained after lyophilization and during the entire storage period [65]. In vitro testing of mRNA-LNPs typically evaluates transfection efficiency, payload distribution, and cellular uptake in relevant cell lines. They can reveal the differences in delivery and efficacy based on lipid composition and formulation parameters before and after the freeze-drying [4,90]. In vivo animal testing, using models such as mice, assesses biodistribution, organ-specific accumulation, and the potency of mRNA delivery [24,90]. Together, these analytical approaches provide a comprehensive understanding of mRNA-LNPs stability over time. These can guide the rational design and optimization of effective freeze-drying preparations.

Additionally, the appearance, cake structure, moisture content, and reconstitution time are critical quality attributes specific to lyophilization. For lyophilized products, a uniform, intact cake indicates successful freeze-drying, whereas collapse or shrinkage may signal process issues. One of the main factors impacting vaccine stability after lyophilization is the residual moisture of the product, which is influenced by the temperature, pressure, and duration of secondary drying. Maintaining a specified range of residual moisture levels is crucial for product integrity. While over-drying is to be avoided, too high residual moisture levels can cause structural collapse during storage and can increase the mRNA degradation rate as the absorbed water provides molecular mobility that can induce chemical degradation and aggregation [21]. Low residual moisture (<1–2%) is necessary for long-term stability [91]. The lyophilized product should be reconstituted quickly and completely, restoring original particle characteristics [65].

## 6. Analytics for the Freeze-Drying Study

In addition to visual inspection, analytical characterization of freeze-dried mRNA-LNPs post-rehydration is essential to evaluate the preservation of key attributes. The reconstituted solution should appear uniform and translucent, like freshly prepared mRNA-LNPs solutions. It is also important that the reconstituted product readily and rapidly dissolves in water or a chosen buffer. After reconstitution, analytical methods such as dynamic light scattering (DLS) for size and polydispersity index (PDI), zeta potential measurements, RiboGreen assays for mRNA encapsulation efficiency, in vitro cell expression study and in vivo animal study are frequently employed [19,20,47]. Electron microscopy techniques such as scanning electron microscopy (SEM), transmission electron microscopy (TEM), and cryogenic TEM (cryo-TEM) are vital for characterizing mRNA-lipid nanoparticles. SEM provides surface morphology, TEM enables visualization of internal structures, and cryo-TEM offers high-resolution images of LNPs in their native hydrated state, which is essential for accurately assessing mRNA encapsulation and distribution within the nanoparticles [43,66]. These analytics confirm the maintenance of LNPs’ integrity and functional performance (Table 5).

## 7. Conclusions

Freeze-drying is now considered a promising strategy to overcome the intrinsic thermal and chemical instability of mRNA vaccines containing LNPs-encapsulated mRNA(s). This review has comprehensively discussed the progress and challenges in freeze-drying mRNA vaccines. Among various lyophilized mRNA-LNPs formulations, the combination of 10% sucrose and 5% trehalose in 10 mM Tris buffer at pH 7.4 emerges as the most optimal formulation. This formulation demonstrated maintaining its physicochemical properties and biological activity (in vitro and in vivo) for at least 12 months at 4 °C. Besides, this formulation showed no significant decline in product quality at ambient temperature for up to 8 h. Another promising formulation includes 9% trehalose with 1% PVP in 20 mM Tris buffer at pH 7.4. This showed excellent physicochemical stability, including the particle size, PDI and encapsulation efficiency at 25 °C for 6 months. Although it is considered a strong candidate for high-temperature storage conditions based on the physicochemical analytics, it lacks immunogenicity data. In addition, another formulation containing 20% maltose in a 5 mM Tris buffer at pH 7.4 demonstrated promising stability. This maintained the in vivo bioactivity for one year at 4 °C and four weeks at room temperature. Overall, the formulations using sucrose and/or trehalose in a Tris buffer system at physiological pH (around 7.4) are most effective in preserving the structural and functional integrity of lyophilized mRNA-LNPs. Mostly, the formulation with 10% sucrose + 5% trehalose in 10 mM Tris (pH 7.4) stands out as the most balanced and practically favorable choice for both physicochemical and biological stability. This also highlights the critical roles of formulation components (disaccharides, sugar alcohols, amino acids, buffers (e.g., Tris, acetate), lipid composition) and process parameters (freezing rate, primary and secondary drying temperature, chamber pressure, thermal properties (e.g., Tg′, Tc)). Key findings from this review demonstrate that the potential usability of freeze-drying will depend on a multifactorial optimization of formulation and process parameters. Besides these parameters, the LNPs’ composition, fill volume, and vial geometry also influence the critical quality attributes (CQAs) of the mRNA-LNPs.

## 8. Future Directions

The integration of digital twin principles and design of experiments (DOE) has been proposed to further improve the freeze-drying process by enabling predictive modelling and systematic optimization of critical process parameters [31,92]. Modelling of the freeze-drying process can simulate heat and mass transfer to anticipate drying behaviour without physical trials. On the other hand, using DOE in freeze-drying helps to identify the significance and interaction of different process parameters (like freezing rate, primary drying temperature, etc.), affecting the critical quality attributes (CQA) of a given mRNA vaccine. These techniques, applied within the Quality by Design (QbD) framework, help to accelerate the process development, reduce experimental workload, and ensure consistent product quality. From a cost-effectiveness standpoint, lyophilization introduces additional process steps, making the process lengthy and expensive. Also, this requires additional operational and capital expenditures, and the need to reconstitute the freeze-dried solid formulations in an appropriate buffer. However, it is worth the investment because it reduces the need for cold chains and prevents the wastage of vaccines. In summary, freeze-drying represents a promising solution to deliver thermostable mRNA vaccines by tackling the challenges of formulation and associated critical process parameters. This process will ultimately strengthen public health impact, pandemic preparedness, and equitable vaccine distribution worldwide.

## Figures and Tables

**Table 1:** Choices of Excipients in the Formulations of Freeze-dried mRNA Vaccines.

Formulations		Protecting Mechanism	Positive Impacts on mRNA-LNPs	References
Sugars	Sucrose	Protective coating prevents mechanical damage, vitrification (formation of a glassy matrix), water replacement (hydrogen bonds), and cryoprotection	Prevents LNPs aggregation, preserves particle size, maintains mRNA integrity, and reduces freeze and dehydration stresses	[11,32,51,54,55]
	Trehalose	Increase formulations’ viscosity, high glass transition temperature (Tg), low crystallization risk, vitrification, water replacement, cryoprotection	Higher Tg’ than sucrose, maintains structural integrity, enhances LNPs resistance to drying	[21,54,56,57,58,59]
	Maltose	Glass matrix formation	Often combined with sucrose, helps prevent structural collapse	[43,60]
Sugar alcohol	Mannitol	Bulking agent, prevents cake shrinkage	Prevents cake collapse, may reduce aggregation, but can crystallize unfavorably	[42,50,61]
Buffer	Tris	Scavenges hydroxyl radicals, stabilizes pH during freezing	Reduces pH shift, improves encapsulation and transfection efficiency, and reduces zeta potential shift	[45,47,62,63,64]
	PBS	Ionic stabilization maintains a stable pH during freezing and drying, but is prone to pH shift in the presence of sodium ions	Common but inferior to Tris, used for its ionic strength, can decrease encapsulation efficiency and stability	[42,45,47,62]
	HEPES	PH buffering, stabilizing effect during freeze-thaw	Helps to maintain LNPs integrity during freeze-thaw cycles and long-term storage	[62]

**Table 2:** Formulation composition of published studies on mRNA vaccine stabilization.

Formulations (*w*/*v*)	Buffer/pH	Reconstitution	Stability	References
10% sucrose 10% maltose	5 mM Tris/ pH 8	Water	Physicochemical properties do not significantly change for 12 weeks after storage at room temperature and for at least 24 weeks after storage at 4 °C	[43]
8.8% sucrose, 2% trehalose, 0.04% mannitol	-	-	The lyophilized mRNA-LNPs were stable at 2–8 °C, and they did not reduce immunogenicity in vivo or in vitro.	[65]
8.7% sucrose	(PBS)	90 μL of nuclease-free water	Optimal O9 mRNA-LNPs could be stored at 4 °C for more than 12 weeks and at room temperature for 4 weeks after lyophilization.	[31]
10% sucrose	PBS/pH 7.4	Deionized water	mRNA vaccines were stably stored in 10% *w*/*v* sucrose in PBS at −20 °C for at least 30 days.	[42]
20% maltose	Tris 5 mM/pH 7.4	300 μL RNase-free water	Lyophilized LNPs retained their in vivo bioactivity at an almost unaffected level for 1 year when stored at 4 °C. Lyophilized LNPs also presented unaltered thermo-stability at room temperature (25 °C) for 4 weeks.	[45]
12.5% sucrose	20 mM Tris/ pH 7.4	400 μL of Tris-, phosphate- or PBS buffer at pH 7.4	Lyophilized mRNA-LNPs preserved their functionality when stored at 4 °C, 22 °C and even at 37 °C for 12 weeks.	[47]
5% sucrose/ 5% trehalose	-	-	5% (*w*/*v*) sucrose or trehalose LNPs stored in liquid nitrogen maintained mRNA delivery efficiency for over three months.	[32]
9% trehalose/ 1% PVP	20 mM Tris/pH 7.4	275 μL RNase-free water	The most promising formulations for storage at higher temperatures were identified as 9% (*w*/*v*) trehalose + 1% (*w*/*v*) PVP, with only a slight increase in size over 6 months at 25 °C, while maintaining PDI and encapsulation efficiency.	[41]
10% sucrose/5% trehalose	10 mm Tris/pH 7.4	Water	Lyophilized mRNA-LNPs can be stored at 4 °C for at least 12 months and at least 8 h after reconstitution at ambient temperature without a significant change in product quality. They also preserved the in vitro immunogenicity in mice, comparable to that of freshly prepared mRNA-LNPs.	[66]
10% sucrose/9% mannitol/1% PEG60	Tris	Water	Dry powder formulation that could maintain the physicochemical properties of mRNA-LNPs after storage at 4 °C for at least two months.	[67]
10% sucrose	-	Nuclease-free water	Lyophilized form of LION/repRNA-CoV-2S with 10% *w*/*v* sucrose, maintained in vivo immunogenicity after 1 week at 25 °C and 6 months at 2–8 °C. Lyophilized LION/repRNA-PyCS vaccine with 10% *w*/*v* sucrose, stored for 12 months at 2–8 °C, demonstrated no loss in immunogenicity.	[68]

**Table 3:** Freeze-drying process parameters of some mRNA vaccine studies.

Freezing (Temperature/Time)	Primary Drying (Temperature/Pressure/Time)	Secondary Drying (Temperature/Pressure/Time)	References
−45 °C/3 h	−25 °C/2.7 Pa/84 h	30 °C/2.7 Pa/5 h	[43]
−40 °C/2 h	−35 °C/10 Pa/24 h	25 °C/5 h	[78]
−40 °C/2 h	−10 °C/16 Pa/17 h	2 °C/6.8 Pa/10 h	[79]
−80 °C/6 h	−50 °C/6 Pa/24 h		[80]
−30 °C/3 h	−25 °C/5–10 Pa/16–18 h	22–27 °C/20 Pa/5 h	[31]
−80 °C	12 h	-	[32,54]
−40 °C/40 min −40 °C/20 min	−30 °C/1 h −20 °C/1 h −10 °C/1 h 0 °C/1 h	10 °C/1 h 20 °C/1 h 30 °C/3 h	[46,81]
−50 °C/5 h	−15 °C/24 Pa/12 h	30 °C/13.3 Pa/7 h	[45]
−40 °C/3 h	−20 °C/13 Pa/10 h	25 °C/5 h	[41]
−20 °C	−30 °C/3 Pa/30 h	25 °C/3 Pa/6 h	[66]
−50 °C/3 h	−50 °C/1 h/27 Pa −40 °C/1 h/27 Pa −35 °C/12 h/27 Pa	30 °C/10 h	[67]
−50 °C/1.5 h	−30 °C/7 Pa/17.5 h	25 °C/7 Pa/1.5 h	[68]

**Table 4:** Key CPPs and CQAs for mRNA-LNPs (Including Freeze Drying).

Critical Process Parameters (CPPs)	Critical Quality Attributes (CQAs)
Lipid composition and molar ratios e.g., ionizable lipid, helper lipid, cholesterol, PEG-lipid)	Particle size and PDI (affects biodistribution, cellular uptake, and dose uniformity)
Buffer type and pH (affects LNPs assembly, mRNA stability, and lyophilization compatibility)	Encapsulation efficiency (% of mRNA encapsulated; impacts potency and dosing)
Lipid: mRNA weight ratio (key determinant for encapsulation and particle stability)	Zeta potential (an indicator of colloidal stability and cellular interaction)
Mixing rate and temperature during microfluidic formulation (impacts LNPs size and uniformity)	mRNA integrity and purity (determines efficacy; assessed by electrophoresis, HPLC)
Freeze-drying (lyophilization) cycle parameters (freezing rate, primary/secondary drying temps and pressures)	Lipid composition/identity post-processing (assures no degradation or phase separation)
Cryoprotectant type and concentration (e.g., sucrose, trehalose; critical for preserving structure)	Appearance and cake structure (e.g., collapse, shrinkage, or uniformity after lyophilization)
Residual moisture content (influenced by secondary drying endpoint)	Moisture content (affects storage stability and reconstitution)
Reconstitution conditions (solvent type, volume, and agitation)	Reconstitution time (speed and ease of redispersion into solution)
Storage temperature and container closure system	Potency/Transfection efficiency (in vitro and in vivo functional activity of mRNA-LNPs)

**Table 5:** Analytical Methods Used for Post-Lyophilization Characterization of mRNA Vaccines.

Property	Analytical Method	Reference Study	Recommended Standard
Particle size	Dynamic light scattering (DLS)	[31,32,42,43,45,47,65]	Between 80 and 110 nm for optimal cellular uptake and biodistribution
Nanoparticle morphology, size, and internal structure	Transmission electron microscopy (TEM) Scanning electron microscopy (SEM) Cryogenic electron microscope Cryo-transmission electron microscopy (Cryo-TEM)	[23,31,41,42,43,45,65,66,67]	Uniform spherical or vesicular structures, depending on the design Between 70 and 90 nm for optimal cellular uptake and biodistribution
Polydispersity index (PDI)	Dynamic light scattering (DLS)	[31,43,45,47,65]	≤0.2 indicates a homogeneous particle population
Zeta potential	Electrophoretic light scattering (ELS) Dynamic light scattering (DLS)	[31,32,42,45,47]	±20 to 30 mV is generally sufficient for colloidal stability and minimal aggregation
mRNA encapsulation efficiency	Quant-it Ribogreen fluorescence assay	[31,43,45,47,65]	≥90–95% is typically targeted for therapeutic efficacy
mRNA concentration	Ribogreen fluorescence assay	[43,45]	Consistency across batches is key; the quantitative threshold depends on dose
mRNA integrity	Capillary electrophoresis	[31,43,45,65]	Intact single bands, degradation products should be minimal or absent
Lipid content	Ultra high-performance liquid chromatography (UHPLC)	[43]	Must match expected lipid: mRNA molar ratios
Residual moisture	Karl Fischer titration	[41]	<1% *w*/*w* is typically recommended to ensure long-term stability and prevent degradation
Visual appearance (cake quality)	Visual inspection (macroscopic evaluation)	[41]	Cake should be uniform, white, intact, without collapse or shrinkage
In vitro transfection efficiency	Luciferase report assay, GFP expression assay	[45,91]	Comparable or improved transfection vs. freshly prepared LNPs
In vitro cytotoxicity	Cell viability assays (CCK-8, MTT)	[65,91]	Usually, >80% cell viability
In vivo immunogenicity	ELISA, HAI assay/titer	[23,43,65]	Robust and comparable immune response to fresh vaccine
In vivo biodistribution	IVIS imaging, fluorescence/RNA quantification in organs	[45,91]	Distribution to the target tissue, with low off-target accumulation

## Data Availability

The original contributions presented in the study are included in the article; further inquiries can be directed to the corresponding author.
